# Detection of circulating tumor cells in blood by shell-isolated nanoparticle – enhanced Raman spectroscopy (SHINERS) in microfluidic device

**DOI:** 10.1038/s41598-019-45629-7

**Published:** 2019-06-25

**Authors:** K. Niciński, J. Krajczewski, A. Kudelski, E. Witkowska, J. Trzcińska-Danielewicz, A. Girstun, A. Kamińska

**Affiliations:** 1 Institute of Physical Chemistry, Polish Academy of Sciences, Kasprzaka 44/52, 01-224 Warsaw, Poland; 2 Faculty of Chemistry, University of Warsaw, Pasteura 1, 02-093 Warsaw, Poland; 3 Department of Molecular Biology, Institute of Biochemistry, Faculty of Biology, University of Warsaw, Miecznikowa 1, 02-096 Warsaw, Poland

**Keywords:** Cancer screening, Nanoscience and technology

## Abstract

Isolation and detection of circulating tumor cells (CTCs) from human blood plays an important role in non- invasive screening of cancer evolution and in predictive therapeutic treatment. Here, we present the novel tool utilizing: (i) the microfluidic device with (ii) incorporated photovoltaic (PV) based SERS-active platform, and (iii) shell-isolated nanoparticles (SHINs) for simultaneous separation and label-free analysis of circulating tumour cells CTCs in the blood specimens with high specificity and sensitivity. The proposed microfluidic chip enables the efficient size – based inertial separation of circulating cancer cells from the whole blood samples. The SERS-active platform incorporated into the microfluidic device permits the label-free detection and identification of isolated cells through the insight into their molecular and biochemical structure. Additionally, the silver nanoparticles coated with an ultrathin shell of silica (Ag@SiO_2_) was used to improve the detection accuracy and sensitivity of analysed tumor cells via taking advantages of shell-isolated nanoparticle-enhanced Raman spectroscopy (SHINERS). The empirical analysis of SHINERS spectra revealed that there are some differences among studied (HeLa), renal cell carcinoma (Caki-1), and blood cells. Unique SHINERS features and differences in bands intensities between healthy and cancer cells might be associated with the variations in the quantity and quality of molecules such as lipid, protein, and DNA or their structure during the metastasis cancer formation. To demonstrate the statistical efficiency of the developed method and improve the differentiation for circulating tumors cells detection the principal component analysis (PCA) has been performed for all SHINERS data. PCA method has been applied to recognize the most significant differences in SHINERS data among the three analyzed cells: Caki-1, HeLa, and blood cells. The proposed approach challenges the current multi-steps CTCs detection methods in the terms of simplicity, sensitivity, invasiveness, destructivity, time and cost of analysis, and also prevents the defragmentation/damage of tumor cells and thus leads to improving the accuracy of analysis. The results of this research work show the potential of developed SERS based tool for the separation of tumor cells from whole blood samples in a simple and minimally invasive manner, their detection and molecular characterization using one single technology.

## Introduction

Circulating tumor cells (CTCs) are living cancer cells separated from the primary tumor, which are responsible for the development and expansion of the metastasis form of cancer^1^.

The time depended evolution and molecular characterization of CTCs in the peripheral blood are crucial and non-invasive sources for the tumor diagnostics, cancer therapy selection, monitoring and prognosis^2–4^.

Since, the tissue biopsies are invasive and expensive, the characterization of tumor based on CTCs analysis in a peripheral blood which act as ‘liquid biopsy’, has attracted recently special attention. Due to the low number of circulating cancer cells at metastases cancer growth which ranges between 1–100 cancer cells per ml of blood^5^ their isolation and detection is still the challenging task and requires the ultrasensitive methods.

The current methods of CTCs analysis usually utilizing both isolation and detection stages, which are usually completed using separated time-consuming technologies and/or expensive equipment. The isolation methodologies include: (*i*) density-based cell separation size, (*ii*) negative selection of leukocytes (using antibodies against hematopoietic cells) or the depletion of leukocytes and erythrocytes, (*iii*) magnetic separation based on magnetic beads modified with antibodies for tumor specific markers; (*iv*) segregation based on size, charge, migratory properties and deformability^6,7^. It should be highlighted that also other types of cells are morphologically comparable to circulating tumor cells therefore the subsequent screening and detection processes are required. The most popular current detection methods involve the following techniques: polymerase chain reaction (PCR), reverse transcription PCR (RT-PCR), fluorescence scanning microscopy, immunofluorescence assays, flow cytometry analysis based on monoclonal antibodies or laser scanning cytometry^7–9^. The reverse transcription PCR enables the examination only a limited number of genes at the same time and also the ability of this technique to detection of multiple cancer markers might be hinder by lacking of appropriate tumor markers expression. Moreover, RT-PCR does not permit the morphological analysis of cells in subsequent test^10^. There is time-consuming and expensive methodology, which makes this technique unsatisfied for routine clinical analysis. Lately, CTCs detection techniques have undertaken a novel approach based on miniaturized, nanomaterials and microfluidic reactions^11^. Utilization of these microscale flow phenomena leads to more efficient, low-cost and high throughput analysis of circulating tumor cells. There are various approaches to isolate and capture CTCs from blood samples in microfluidic setup including size based isolation^12–14^, dielectrophoresis^15–24^, pinched flow^25–27^, or ultrasonic resonances^28,29^. For example, recently Park and *et al*.^30^ presented an efficient isolation of CTCs in microfluidic chip using a thiolated ligand-exchange reaction with gold nanoparticles (immune-affinity approach). This approach gets closer to the ‘liquid biopsy’ but still requires the time-consuming and complex NHS-strategy of antibodies immobilization on AuNPs including problems with nonspecific adsorption of antibodies. Moreover, the CTCs were finally identify using immunofluorescence techniques (cell-surface receptors were labelled with phenylindole based dyes). Although the accuracy of immunofluorescence is well established this technique still suffers from well-known drawbacks: quenching of the fluorescence signals at excitation, many false positives caused by nonspecific absorption of antibodies. The ‘gold standard method’ of CTCs detection which satisfy high specificity, sensitivity and accuracy still do not exist. Here we present a new strategy based on surface-enhanced Raman spectroscopy (SERS) to detect CTCs from blood samples in microfluidic chip. In contract to fluorescence technique the SERS gives strong, narrow and sharp fingerprint-like signature, which is easily distinguishable even in very complex biological media. The SERS effect results from molecules adsorbed onto specially designed metallic surface (usually Ag and Au and their alloys). The mechanisms of SERS enhancement are attributed mainly to the electromagnetic filed (EM) enhancement based on resonant interaction of light with the surface plasmons in the metal and also to the chemical (CT) mechanism due to charge-transfer transition between a SERS substrate and an adsorbed molecule^31^. Average enhancement factors are about 10^4^–10^6^ but the values about 10^14^ even with the possibility of single molecule detection has been also achieved^32^. Such huge enhancement factor guarantees the SERS performance for variety of ultrasensitive analysis. In addition, the SERS technique provides nondestructive, reliable, rapid, label–free analysis with elimination of expensive reagents and time-consuming sample preparation steps. All these advantages lead to an increase in the practical applications of this technique especially for biological materials - from single macromolecules to cells and microorganisms. Recently, the capabilities of SERS technique in tumour cell identification have been discussed^33^. These approaches exploiting the SERS imaging technologies using SERS probes based onto modified Ag or Au nanoparticles (NPs). Jun *et al*.^34^ developed the silica-encapsulated magnetic nanoparticles (MNPs) with unique properties for cancer cells targeting and identification. SERS nanoparticles modified with epidermal growth factor (EGF) antibody have been used as a targeting ligand to successful CTCs detection even in the presence of white blood cells^35^. In 2014, Shi *et al*.^36^ reported detection of cancer cells based on the designed folate-conjugated magnetic nanoparticles and SERS probes. All these methods require extra labelling strategy for preparation e.g. Raman reporter encoded nanoparticles, tumour cells prelabeled with nanoparticles or nanoparticles modified with peptides. However, it should be highlighted that in all these method the magnetic tapping stage may give the false-positive signals and introduce the additional errors in analysis^34^. Recently, Krafft *et al*.^37^ presented a microfluidic device combined optical tweezers to gather the normal Raman spectra of single red blood cells. However, this method suffers from quite weak normal Raman spectra of analyzed cells and authors suggest that SERS technique can be used in the future for the signal amplification.

In our approach we offer a single technology that is optimal for CTCs isolation, detection, and molecular analysis. We have elaborated the novel type of SERS platform based on photovoltaic device covered with thin layer of silver (Ag/PV). This SERS –active substrate ensures high sensitivity and reproducibility of recorded SERS signals. Additionally, to improve this sensitivity the SHINs (shell-isolated nanoparticles) in the form of silica coated silver nanoparticles (53 ± 11 nm Ag@SiO_2_ 2–5 nm) were injected into microfluidic chip.

Separation of CTCs from whole blood has been performed in the size-based inertial microfluidic system. This approach offers numerous of advantages, including: (i) efficient isolation of cells without pre-labelling or complex preparation of samples, (ii) minimalized volume and costs of analytes, (iii) reduced time of analysis, and (iv) increased sensitivity and specificity via integration with SERS-active nanostructures and shell-isolated nanoparticles.

Moreover, multivariate statistical method as principal component analysis (PCA) was applied to analyze the SERS data in the terms of: (*i*) distinguishing the spectral differences among the studied cells; (*ii*) extracting individual biochemical information from SERS features of each particular type of cell, and (*iii*) developing the models, which permits the simultaneous differentiation and classification of CTCs in complex clinical samples. To the best of our knowledge the combination of solid SERS – active platform with shell-isolated nanoparticles has not be used in circulating cancer analysis, including microfluidic techniques.

Our method can improve selectivity, sensitivity, and, most importantly, significantly reduce the time needed to perform the analysis in comparison to nucleic acid-based techniques, microarray, sequencing, and/or protein-based techniques. In our studies the efficient capturing and accurate detection of renal carcinoma cell (Caki-1), cervical carcinoma cell line (HeLa) in the whole blood samples were performed. Moreover, the proposed SERS-based CTCs identification was done within the microfluidic devices and supported with chemometric multivariate analysis.

## Experimental section

### Cells cultivation and preparation

The renal cell carcinoma (Caki-1) and epitheloid cervical carcinoma (HeLa) cell lines were used for experiments. The Caki-1 cell line purchased from ATCC was a kind gift from Professor Anna Czarnecka (Department of Oncology, Military Institute of Medicine, Warsaw, Poland). The HeLa cell line came from the European Collection of Cell Cultures (ECACC) and was supplied by Sigma-Aldrich (St. Louis, MO, USA).

The renal carcinoma Caki-1 and epitheloid cervical carcinoma HeLa cells were cultured in RPMI-1640 and DMEM media, respectively. Both media were supplemented with 10% FBS, streptomycin (100 μg/ml) and penicillin (100 U/ml). The cell cultures were cultivated at 37 °C, in humidified atmosphere of 5% CO_2_. During experiments the cancer cells were cultured in 25 cm^2^ cell culture flasks. After reaching 80% of confluence, the cells were washed with PBS buffer and trypsinized (0.05% trypsin, 0.02% EDTA solution). Subsequently, the cells were collected, centrifuged at 250 × g for 5 min at room temperature, resuspended in PBS and centrifuged again. After last centrifugation cells were resuspended in 20 μl of PBS and stored on ice. All the media and chemical reagents were obtained from Sigma-Aldrich (St. Louis, MO, USA). The initial concentration (after cultivation step) of cancer cells in PBS was ca. 0.44 × 10^6^ cells/ml and was further spiked into the human whole blood diluted with PBS to the 20% of hematocrit level with the final ratio 1:800 cancer cells to blood cells (comparable to clinical samples).

The human blood samples derived from 10 healthy volunteers, available by courtesy of Regional Blood Center (Warsaw, Poland) were used in our studies. An informed consent was obtained from all subjects (healthy volunteers). The performance of all experiments was in agreement with the institutional guidelines and relevant laws and approved by the Ethics and Bioethics Committee of Cardinal Stefan Wyszyński University in Warsaw.

### Fabrication of photovoltaic-based SERS-active platform

**Figure 1:**
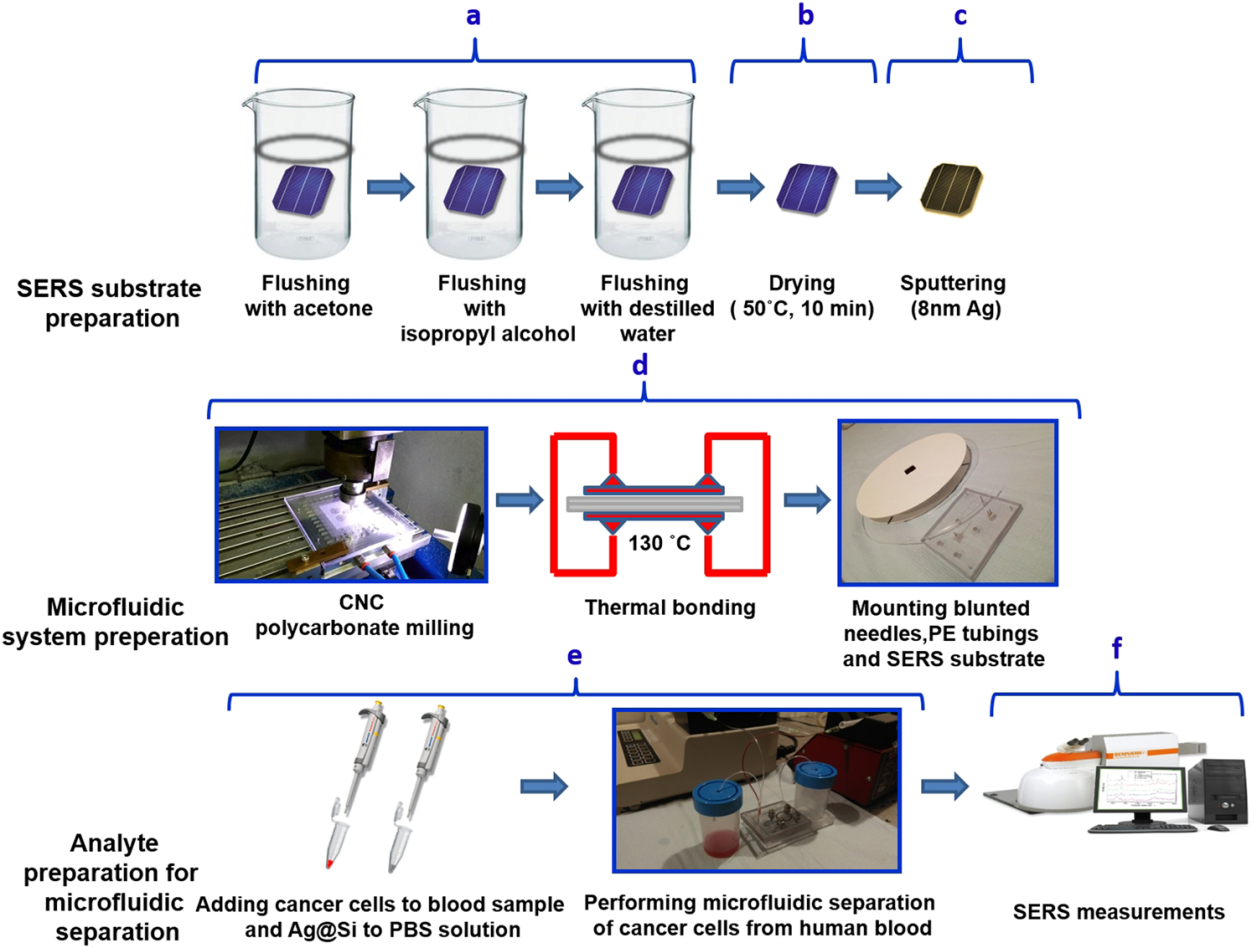
Scheme of the preparation of SERS substrate, microfluidic system, analyte and measurement. Main steps involve cleaning (**a**), drying (**b**), sputtering of thin layer of silver (**c**), the process of preparing microfluidic system (**d**), analyte and buffer preparation followed by the separation of tumor cells (**e**) and finally the measurement takes place (**f**).

**Figure 2:**
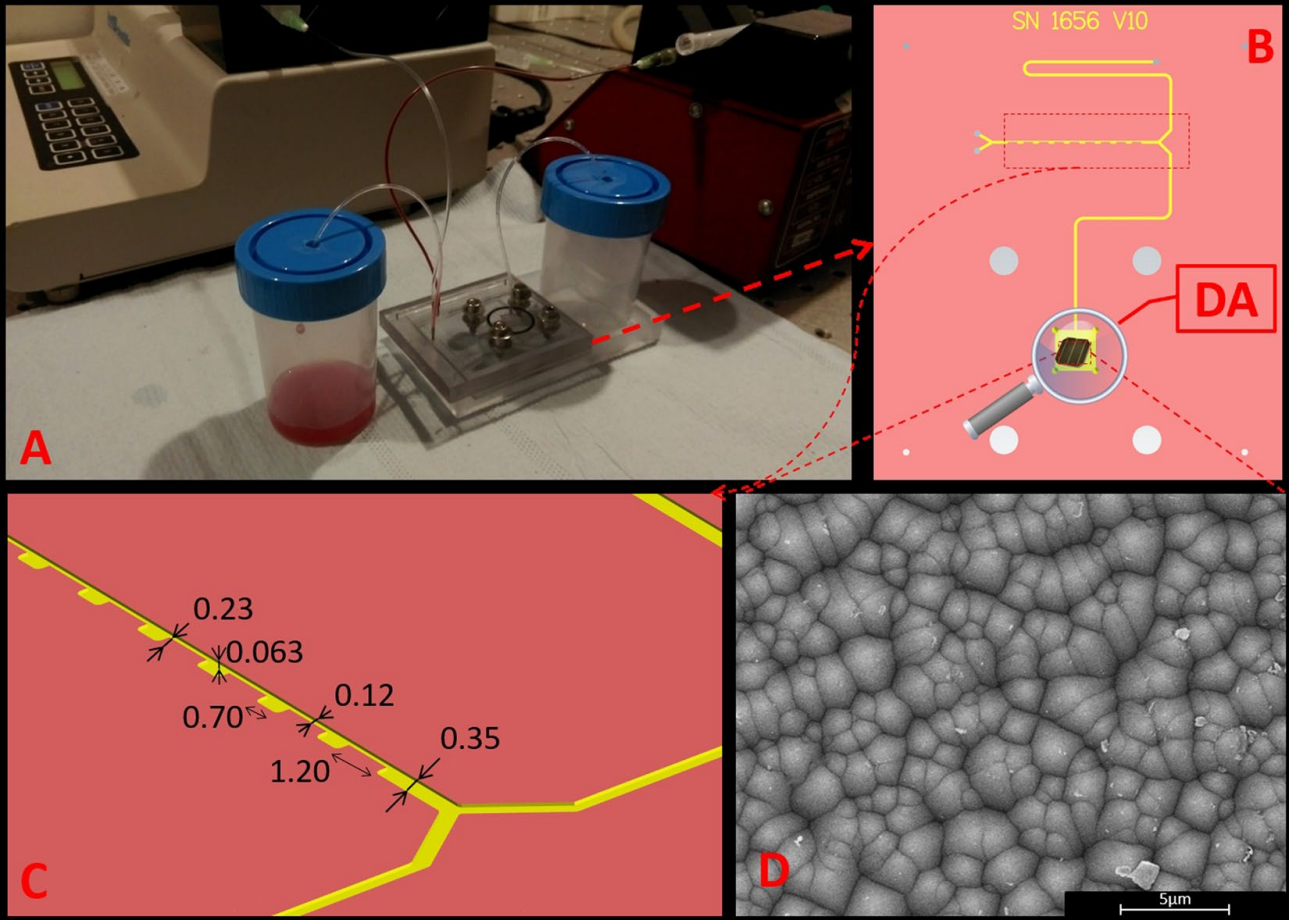
(**A**) Setup used for CTCs separation from whole blood, (**B**) top view of microfluidic device, (**C**) cross-sectional view of microchannel (dimensions in the figure are given in millimeters), and (**D**) SEM image of SERS-active platform incorporated into the detection area chambers (DA) of microfluidic device.

General scheme of preparation of SERS-active platform and measurement of the sample is shown in Fig. 1. Polycrystalline, silicon-based photovoltaic panels have been provided to us thanks to the courtesy of from Bruk-Bet Solar Tarn´ow, Poland as a post-production rest. Photovoltaic sample at 40 × 40 mm was placed in baker filled with acetone. The first step was sonication for 10 minutes in ultrasonic bath at temperature 50 °C (Fig. 2A). Then the acetone was exchanged and the step was repeated. After 10 minutes the baker was filled with isopropyl alcohol and the sample was sonicated for 10 minutes at temperature 50 °C (Fig. 2B). Then the sample was sonicated for 10 minutes in distilled water (Millipore) at ambient temperature (Fig. 2C). Cleaned photovoltaic device was then dried for 30 minutes at 50 °C (Fig. 2D) and placed in sterile Petri dish or immediately placed in Physical Vapor Deposition (PVD) device and sputtered with layer of silver (Fig. 2E). The prepared SERS platform is ready for use. In order to get acquainted with their extensive characteristics, we recommend to get familiarized with our previous work^38^.

### Sputtering of thin layer of SERS active metal

The PVD device (Leica, EM MED020) was applied to sputter the Ag directly on the PV surface. No adhesion layer, i.e. chromium or titanium, was used between photovoltaic surface and the Ag layer. The thickness of the Ag layer was set to 8 nm for all analyzed samples. The sputtering conditions: current −25 mA and the pressure −10^−2^ mbar.

### Synthesis of SHINs

Silver nanoparticles were prepared using slightly modified standard Turkevich’s method of the synthesis of gold and silver nanoparticles^39^. To synthesize silver nanoparticles, 125 ml of 10^−3^ M aqueous solution of AgNO_3_ was placed in a round-bottom flask and heated to boiling under stirring. In the next step, 10 ml of a 1% solution of sodium citrate was added and the obtained mixture was kept boiling for 30 min under stirring. Then, the obtained sol was cooled to room temperature and allowed to age overnight in dark before deposition of the silica layer.

Silica layer on silver nanoparticles has been formed by the decomposition of tetraethyl orthosilicate. In the first stage, the sol of nanoparticles was centrifuged at 5 × 10^3^ g for 15 min. After centrifugation the supernatant was carefully removed, and the precipitate was redispersed in a smaller amount of water (to increase concentration of nanoparticles 10 times). Then, 1 ml of concentrated aqueous sol of metal nanoparticles was introduced to 9 ml of isopropanol under vigorous stirring and 0.19 ml of 25% ammonia aqueous solution and 4 μl of tetraethyl orthosilicate have been added to the aqueous/isopropanol sol of Ag nanoparticles. The mixture were stirred for 30 min at 28 °C. After that time the obtained sol of silica-covered nanoparticles was concentrated by centrifuging for 15 min at 5 × 10^3^ g, the supernatant was poured out and the precipitate was redispersed in water. This cleaning procedure was repeated four times.

### Fabrication of a microfluidic chip

The microfluidic chip has been designed using MasterCAM software and then micromachined with a computer numerical-controlled (CNC) milling machine (ErgWind, type MFG4025P) in a 5 mm polycarbonate (PC) slab (Bayer).

The milled channels had the narrowest sections, 1.20 mm in length, 0.12 mm wide and 0.063 mm depth. The wider sections were 0.70 mm long and 0.35 mm wide. To join milled and plain PC slabs we pressed them together at high temperature (T = 130 °C) for 30 minutes. A high precision syringe pump system (Harvard Apparatus Pump Series, MA, USA) was used for automated control of flow. To inject the analyte and buffer into the system and collect the separated fractions, holes with a diameter of 0.8 mm were drilled in appropriate places in the plates. Blunt ended needles with an outer diameter of 0.8 mm were installed in the holes. Standard polyethylene (PE) tubings with an inner diameter of 0.8 mm were used for interconnection of the chip at the syringe pump. The SERS-active platform based onto photovoltaic array was placed onto the detection area chambers (DA) of the microfluidic chip (Fig. 2C) to record SHINERS signals from this point. The detection points are open to air and the recorded SHINERS signals are not affected by the material of the microfluidic device.

## Instrumentation

### SERS measurements

A Renishaw inVia Raman system was equipped with a 785 nm diode laser as excitation source. The light from the laser was focused on the measured sample with a × 50 microscope objective, NA = 0.25. The beam diameter was approximately 2.5 µm. The spectroscopic maps were acquired by collecting SERS spectra over the previously defined range (36 × 36 μm^2^) at each point on a grid with 3 μm spacing using an automated microscope stage. Typically, 40 SHINERS spectra for each cell types were acquired for 20 s each, by using this mapping mode. The laser light measured at the sample gives power at about 2.5 mW.

### SEM measurements

SEM images were acquired on the FEI Nova NanoSEM 450 instrument operating at an accelerating voltage of 10 kV.

## Chemometrics

Principal component analysis is a multivariate procedure that reduces a multi-dimensional data-set (numbers of observed variables) to a set of new meaningful principal variables. PCA method assignments the data into a new coordinate system crossed by the orthogonal and uncorrelated principal components (PCs). The calculated PCs extract the significant information from the whole introduced data sat.

The PCA analysis over SHINERS data allow to define the most appreciate region or marker bands in order to evaluate spectral differences or similarities among all studied samples that are essential for group separation. Additionally, these most significant diagnostic vibrations (marker bands) in the spectra can be indicated by plotting the wavenumber (Raman shift) as a function of loadings.

All recorded SHINERS data were optimized for principal component analysis (PCA) using following steps. Firstly, the spectra were smoothed with Savitzky-Golay filter, the background corrected (concave rubber band correction; no. of baseline points 34; of iterations 10, no.), and normalized using OPUS software (Bruker Optic GmbH 2012 version). All the data were introduced to PCA analysis using the commercial Unscrambler@ software (CAMO software AS, version 10.3, Norway). The PCA was completed base onto the NIPLAS algorithm, validation (random with 20 segments), significance 0.05 and the 120 number of samples (SHINERS spectra).

In present studies the PCA calculations were performed on three SHINERS data sets: blood cells, HeLa, and Caki-1 cells to extract the most relevant variables among the studied samples.

## Results and Discussion

### SERS substrate characterization

The intensity of the Raman spectra can be amplified by the use of appropriate designed SERS–active surface. The morphological features like the type, size and shape of nanostructures building the SERS platform affect the surface plasmon resonance thus influence the SERS effect. The strongest SERS enhancements are seen for coinage metals: Ag, Au, and Cu, which support surface plasmon resonances in the UV-vis and near-IR visible spectral regions. The desired SERS-active structures should reveal a high SERS signal enhancement defined by enhancement factor (EF), physical and chemical stability, high reproducibility of recorded signals and the possibility to be produced by cheap and reptile technique. In recent years, various methods have been explored to produce and optimize the properties of SERS platforms in the terms of size, shape, and composition of used plasmonic nanostructures^40–43^. Even though a many efficient SERS supports have been developed^44,45^, the some disadvantages of them associated e.g. with low stability with time, upscale and expensive production, and inability to perform *in situ* measurements and sample mapping still exist. Therefore, the new production methods of SERS support still should be develop in order to introduce SERS technique to practical biomedical and analytical applications. In our research group we have shown that polymer mats covered via PVD technique with gold or gold-silver alloy may work as the SERS substrate for the identification of bacterial cells from environmental and human fluid samples^46,47^.

**Figure 3:**
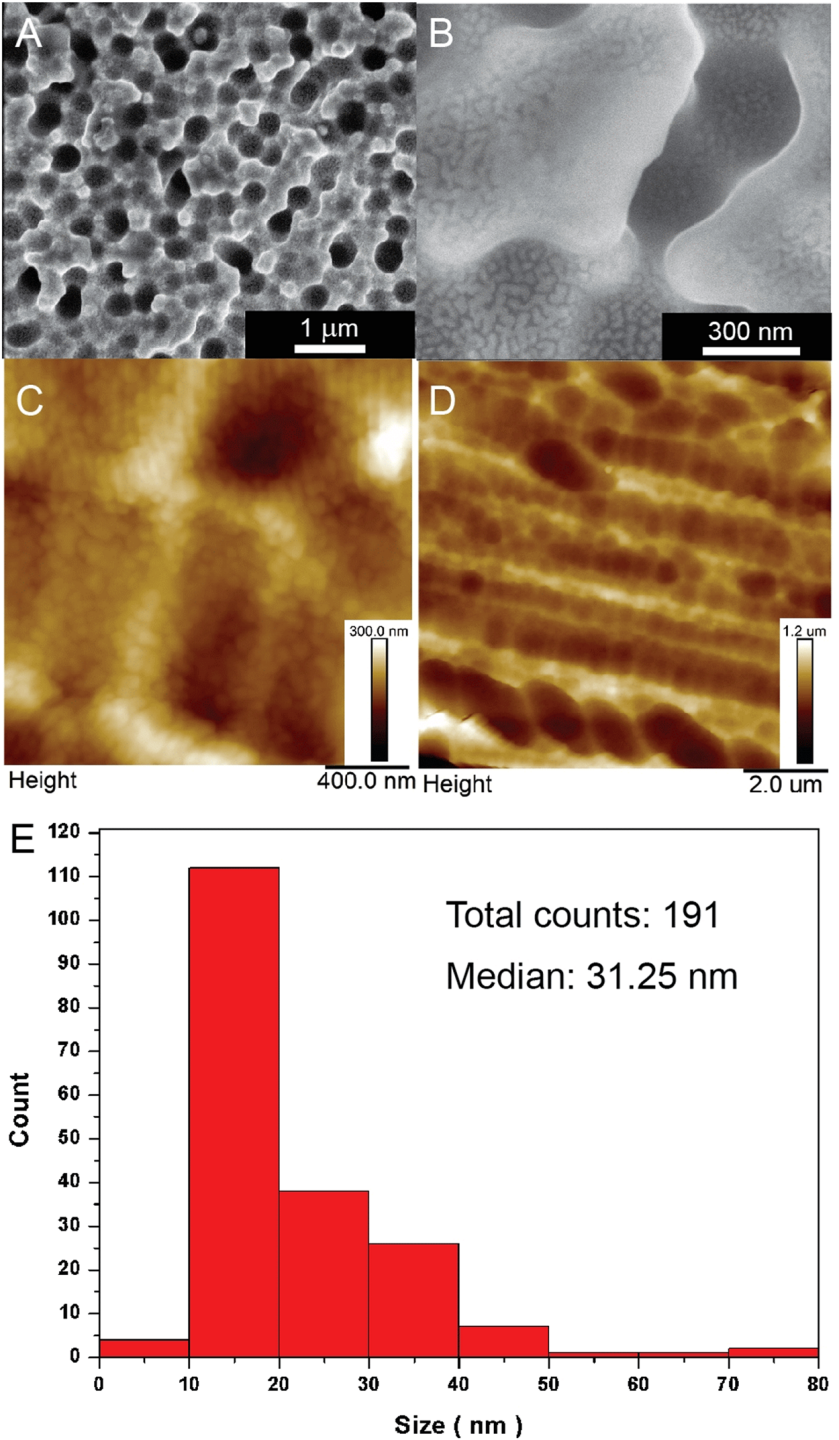
SEM (**A**,**B**) and AFM (**C**,**D**) images at different magnifications of Ag/PV SERS-active platforms sputtered with 8 nm layer of silver via PVD technique. (**E**) presents the histograms of the size of the silver objects on the surface of the PV based substrates.

In this study we have applied the photovoltaic based SERS platform (Ag/PV) for the label-free analysis of circulating tumor cells from blood samples. As was mentioned above, the PV arrays were subsequently covered with silver by PVD process to achieve the SERS activity. The SERS efficiency (sensitivity, selectivity, and reproducibility) was demonstrated for *p*-aminothiophenol (*p*-ATP), which is commonly used a standard probe molecule and has been described in details in our previous article^38^. The morphology of these SERS-active substrates was visualized by the atomic force microscopy (AFM) and scanning electron microscopy (SEM) techniques and is presented in Fig. 3.

Figure 3C,D presents the AFM pictures at different magnifications of fabricated SERS-active platform. AFM image at high magnification shows uniformly placed silver nanostructures. The degree of surface roughness RMS (root mean square value) of PV samples was determined using AFM and equals 120 ± 6 nm. The SEM images (Fig. 3A,B) also confirm that the developed method of SERS-active fabrication offers a homogeneous coverage of the cones surface with the layer of silver. To quantitatively asses their size distribution we applied image analysis. The histogram of the size of the silver nanostructures is depicted in Fig. 3E. The median size of the objects on the surface is below 40 nm, with optimal size of nanostructures (40–60 nm) for the LSPR resonance. The silver nanostructures were densely and homogeneously deposited onto the Ag/PV surfaces, which ensure the strong and reproducible SERS responses.

The developed SERS-active substrate exhibits the high enhancement factor (EF), high stability and reproducibility of the recorded spectra^38^. For *p*-aminothiophenol the enhancement factor (EF) of the Raman signal on a Ag/PV surface was estimated as high as 10^6^. The SERS measurement reflects also the excellent reproducibly of spectroscopic signals (Fig. S2). The reproducibility was validated by quantifying the intensity variation of the band at 1078 cm^−1^ in *p*-ATP SERS spectra. The calculated relative standard deviation equals 3.6%.

Such designed SERS platform has been combined with the microfluidic device (Fig. 2) to perform Raman analysis of renal cell carcinoma (Caki-1) and cervical carcinoma (HeLa) circulating tumor cells spiked into the human whole blood diluted with PBS to the 20% of hematocrit level with the final ratio 1:800 cancer cells to blood cells (comparable to clinical sample).

### Shell-isolated nanoparticles (Ag@SiO_2_) characterization

**Figure 4:**
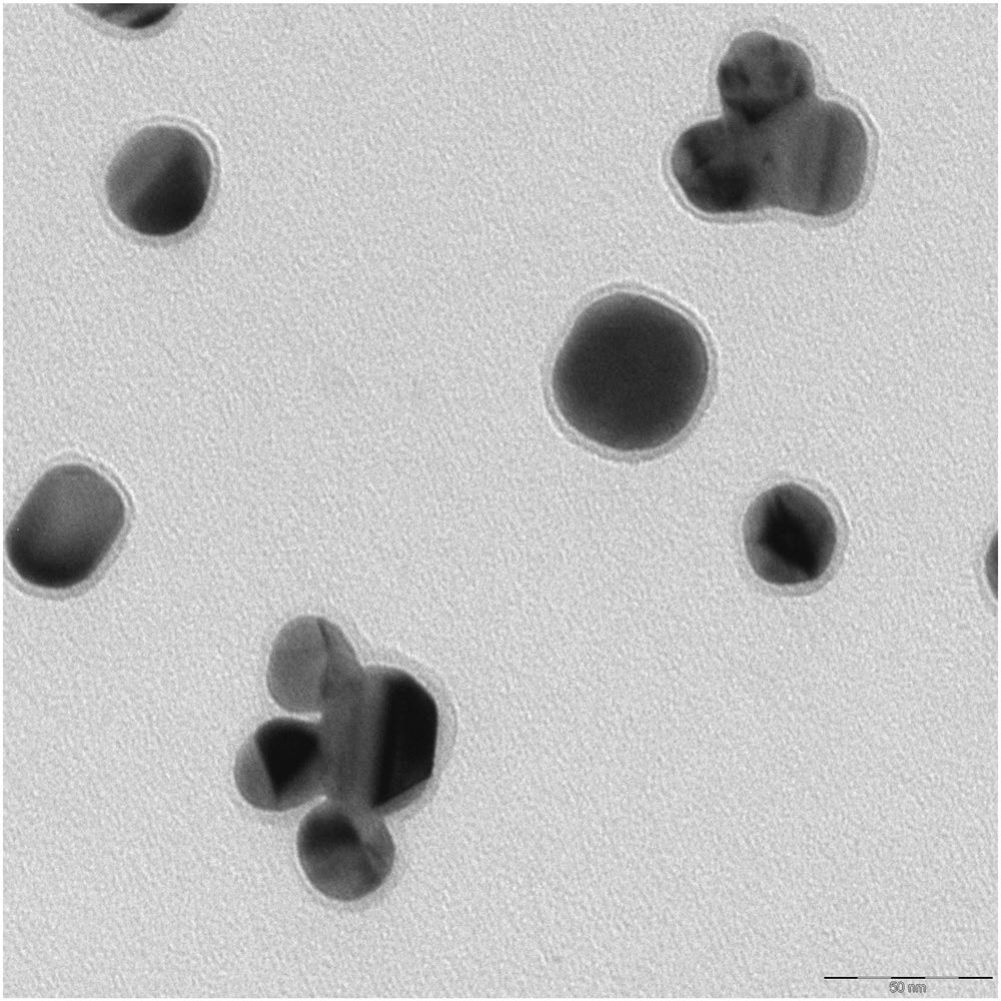
TEM micrographs of Ag@SiO_2_ nanostructures.

Analysis of shell-isolated nanoparticles were performed via transmission electron microscopic (TEM). The average diameter of obtained silver nanoparticles determined from the analysis of 500 TEM images is equal to 53 ± 11 nm. Silica layer on silver nanoparticles has been formed by the decomposition of tetraethyl orthosilicate. Figure 4 shows example TEM image of obtained Ag@SiO_2_ nanoparticles. As can be seen from this Figure the thickness of the formed SiO_2_ layer is about 2–5 nm.

### Cancer cells isolation

**Figure 5:**
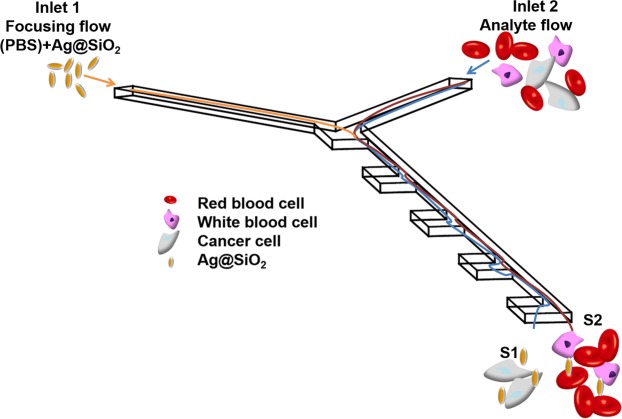
Schematic view of cancer cell isolation from whole blood.

The efficient cell isolation is an essential step in many medical studies and analytical tests. The application of microfluidic-based systems for cells separation offer numerous of advantages^48^ increasing the potential for point-of care diagnostic. In this study we present the microfluidic device with incorporated SERS-active platform, which exploits the inertial size-based separation of CTCs cells from whole blood samples. The proposed strategy for HeLa and Caki-1 cell lines isolation utilizes combination of two effects: (i) inertial lift force and (ii) Dean flow^49^ (see Fig. 5). Additionally, based on the cellular size of CTCs (20–30 µm), which is larger in comparison to diameter of other blood cells (2–15 µm)^50,51^, the size-based separation can be performed. The chip was designed to allow, besides CTCs isolation, also their spectroscopic analysis and identification. Therefore, the special arrangement and the flow rates of blood and buffer streams have been tailored to determine the optimal separation of CTCs and SERS-based detection. To evaluate the separation strategy of our system, in the first step, the studied cancer cells (HeLa, Caki-1) were spiked into the human whole blood diluted with PBS to the 20% of hematocrit level with the final ratio 1:800 cancer cells to blood cells (comparable to clinical samples^52^). Then, such mixture of the cancer cells and blood cells was introduced via inlet 1 and focused by mixture of PBS fluid with SHINs (shell-isolated nanoparticles) injected via inlet 2 (Fig. 5). As was mentioned above, the separation of CTs from other blood cells has been performed using the inertial size-based microfluidics device. This hydrodynamics separation technique has been experimentally confirmed and theoretically analysed^49,53^. The cancer cells movement is mainly caused by the inertial lift force, therefore these large cells move towards sidewall S1 (Fig. 5). The smaller blood cells, which movement is mainly influenced by Dean flow at each entrance of the concentration region, flow towards sidewall S2 (Fig. 5). The stream of isolated CTCs pass through the optical chamber with incorporated SERS-active platform and were finally analyzed by recorded SHINERS spectra.

In the proposed method there is no need to use the separate techniques to perform filtration, enrichment and examination of circulating in blood tumor cells. This is the main advantage of proposed method. Additionally, by combining these three basic steps in detecting cancer cells in one single process the transfer of cells from one place/method to another is eliminated. Therefore the proposed strategy prevents the contamination of sample and cells decomposition, and leads to improving the accuracy of analysis. Moreover, the chip-based detection reduces the time of analysis and minimalizes the quantity of reagents.

### SHINER investigations of circulating tumor cells

Label-free SERS based detection can differentiate the cancer cells from normal cells via quantitative and qualitative differences in amino acids, proteins, nucleic acids, lipids, providing the characteristic SERS fingerprinting. Raman spectra allow not only to distinguish cancer cells from normal blood cells but also to identify different cancer cell lines^10,54^ with high specificity and non-destructivity. As the amount of single CTCs in peripheral blood is rare^50^, the highly-efficient cell enrichment and single cell capture are essential to screen target cells. Therefore, in the next step the spectroscopic fingerprints of just captured CTCs are recorded to perform detail molecular analysis and identification of studied cells.

In order to collect the reference spectra of all studied cells (Caki-1 and HeLa) the SERS measurements were performed directly from pre-cultures. Fig. S3 in Supplementary Materials presents the obtained SERS spectra. Table 1 presents the main SERS bands observed in analysed cells and the corresponding bands assignments.

**Table 1:** Assignment of SERS bands depicted in Fig. 6^66,67,72–75^.

Observed SERS band (cm^−1^)	Protein	Lipids	Nucleic acid
650–658	Tyr (C-C twist)		
725–730	Trp	C-N head group choline (H_3_C)_3_N+	A
789			PO_2_ symm
827	Structural protein modes of tumors		
850	Tyr, Pro		
925	C-C str alpha-helix, Pro, Val		
960	CH_3_ def	CH_3_ def	
1002	Phe		
1030–1032	Phe	CH_2_CH_3_ bending modes of lipids	
1090	C-N stretch	CC str chain, C-O str	PO_2_ symm
1125	C-N str bk	porphyrin	
1170–1172	Tyr C-H in plane		T
1217	C-C_6_H_5_ str in phenylalanine tyrosine and Amide III (beta sheet)		
1246	Amide III		
1267–1270	Amide III (random coil)	=CH def	
1319	CH_3_ def, collagen	CH_3_CH_2_ twist	G
1340			A,G
1417	C = C stretching in quinoid ring		
1450	structural protein modes of tumors		
1552			A,G
1585–1600	Phe, Tyr		
1613–1618	C≡C str of Tyr and Trp		
1647–1665	Amide I	C = C str	

**Figure 6:**
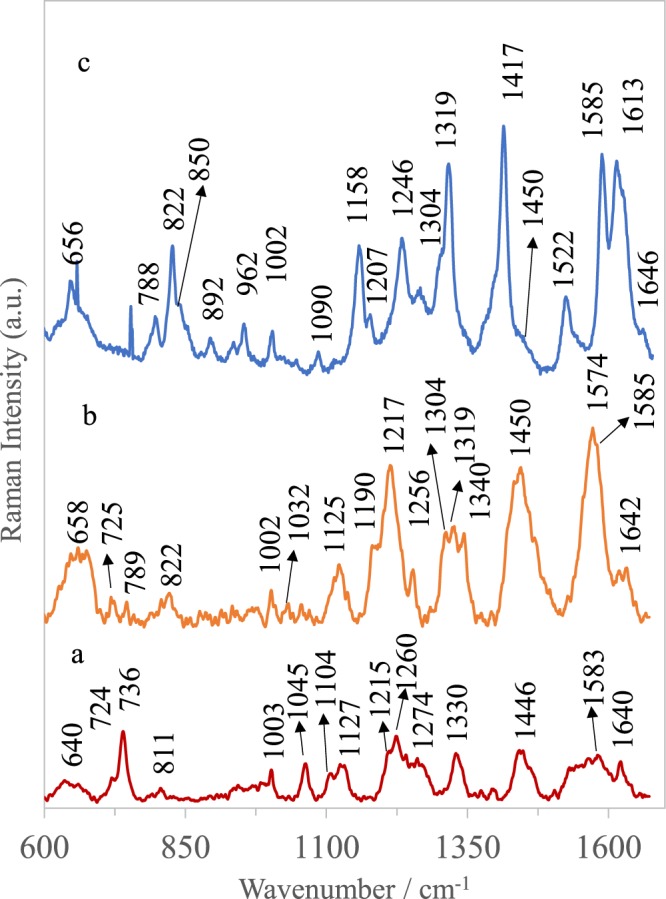
Averaged and normalized SHINERS spectra of (**A**) blood cells, (**B**) Caki-1, and (**C**) HeLa cells recorded on Ag/PV SERS platform in microfluidic device with SHINs (shell-isolated nanoparticles).

Figure 6 reveals the examples of SHINERS spectra of the cells captured and detected from blood samples using the microfluidic device according the procedure presented in Figs 2 and 5. All the spectral fingerprints depicted in Fig. 6 matched with the reference data in Fig. S3. As can be observed in Fig. 6, the SHINERS spectra reveal common spectroscopic features characteristic to the constituent of the eukaryotic cell^55^: nucleic acid, proteins, and lipids. The shoulders around 1256 cm^−1^ and 1600 cm^−1^ ate assigned to amide I bands. Aromatic amino acid contributions appeared around 1002 cm^−1^ (phenylalanine) and 656 cm^−1^ (tyrosine). The vibrational modes of nucleic acid are present at 789 and 1090 cm^−1^. As can be observed, all cells reveal also their own individual spectral signatures, e.g. the band at 1417 cm^−1^ corresponded to adenine and guanine, nucleic acid nucleotides and the band at 1522 cm^−1^ associated with carotene^56^ can be seen in Caki-1 cells, but not in the HeLa. The relative intensities of some bands can also serve as the way of cell differentiation. In the SHINRS spectrum of Caki-1 appeared very intensive band at 1450 cm^−1^ and week band at 822 cm^−1^ both corresponded to the structural protein modes of tumors^40^. These two bands are also present in the SHINERS spectrum of HeLa cells but with changed relative intensities (Fig. 6a,b). All these dissimilarities enable identification of circulating tumor cells.

In the SHINERS spectrum (Fig. 6c) of blood cells (leucocytes, erythrocyte) captured and measured after CTCs filtration (see Figs 2 and 5) appeared many bands typical for porphyrin (1215, 1446, 1583 cm^−1^), as well as signals assigned to globin vibrations (640, 724, 1003, 1260, and 1330 cm^−1^), and some others, which may include contribution from proteins and lipids (1104, 1127, 1260 cm^−1^)^57–60^.

The most prominent bands appeared at 640 cm^−1^ (globin and cellular components, cysteine)^61^, 736 cm^−1^ (thiocyanate)^54^, 1003 cm^−1^ (C-C of phenylalanine)^62^, 1045 cm^−1^ (in plane ring CH deformation mode of phenylalanine), 1127 cm^−1^ (C-N, C-C stretch in plane of protein)^63^, 1260 cm^−1^ (proteins, lipids: amide III), 1330 cm^−1^ (globin and cellular components), 1446 cm^−1^ (globin and porphyrin)^62^. The majority of SHINERS features of blood are assigned in Table S1.

Spectroscopic data revealed, that the tumor cells can be distinguished from blood cells using bands at 656 cm^−1^ (C-C twisting mode of tyrosine), 1158 cm^−1^ (CH_2_CH_3_ bending modes of lipids)^64^, 1319 cm^−1^ (CH_3_ deformation mode of collagen), 1417 cm^−1^ (C = C stretching in quinoid ring), 822 cm^−1^ and 1450 cm^−1^ (structural protein modes of tumors)^65^. The peak at 1450 cm^−1^ is assigned to overlapping asymmetric CH_2_ bending and CH_2_ scissors vibrations. Phospholipids, elastin, and collagen were also recognized to have a peak in this region^66^. These differences reflect the changes in biochemical pattern between normal blood cells and cancer cells as the result of abnormal metabolism associated with cancer transformation.

It should be noted that, the SHINERS spectrum of blood cells is evidently less intensive in comparison to SHINERS spectra of cancer cells. It is related with additional enhancement of Raman signals of separated CTCs via introduced shell-isolated nanoparticles (Ag@SiO_2_), which are influenced via the same forces as only CTCs cells (see Figs 2 and 5).

**Figure 7:**
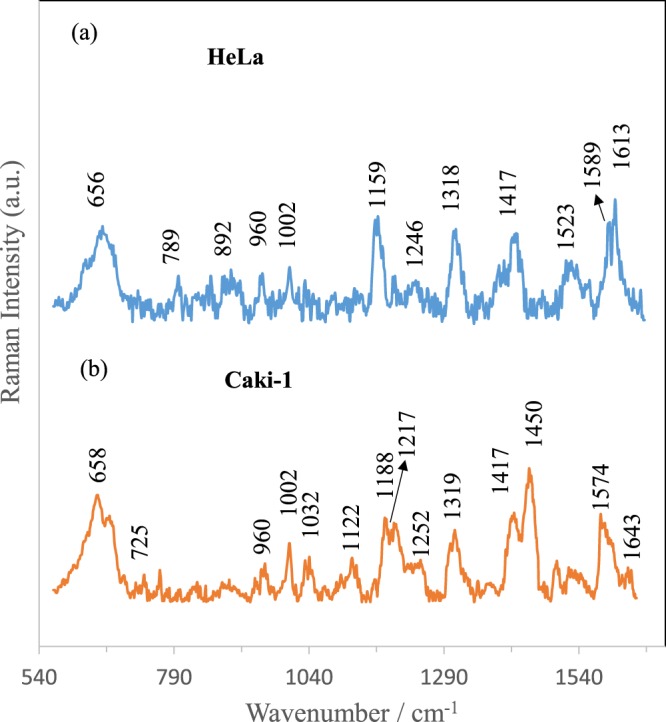
Averaged and normalized SERS spectra of (**A**) HeLa and (**B**) Caki-1, cells recorded on Ag/PV SERS platform in microfluidic device without SHINs (shell-isolated nanoparticles).

In this studies, the impact of shell-isolated nanoparticles onto SERS sensitivity of isolated cancer cells has been also examined. Figure 7 presents the SERS spectra of HeLa and Caki-1 cells isolated according to the same scheme presented in Figs 2 and 5 but without introducing the SHINs (shell-isolated nanoparticles) into the microfluidic chip.

As can be seen from comparison of SERS data presented in Figs 6 and 7, introducing of SHINs gives an opportunity to enhance the sensitivity of developed SERS-based method of circulating tumor cells identification.

In order to check the significance of obtained results and to show the applicability of presented SHINERS strategy in cells differentiation, the reproducibility of the SHINERS signals of Caki-1, and HeLa cells was calculated. The calculations were done for 100 SHINERS spectra measured from the same SERS substrate. The calculations of relative standard deviation (RSD) were performed for the strong signals at 658 and 1613 cm^−1^ and the achieved result was 8.5% and 7.2%, respectively (Supplementary Materials, Table S2).

This excellent reproducibility is due to applying the microfluidic device that enables separation, enrichment and subsequent monitoring of isolated tumor cells. Moreover, the proposed architecture allows the collection of SHINERS signals from one spot at SERS-active platform during the whole process of detection.

Finally, to determine the most diagnostically significant SHINERS features and the effective classification among studied cells the statistical analysis was carried out. The principal component analysis (PCA) was applied as an effective technique that allows spectral similarities or differences observation by delivering the ability to categorize SHINERS spectra that otherwise are hardly distinguishable via empirical examination.

**Figure 8:**
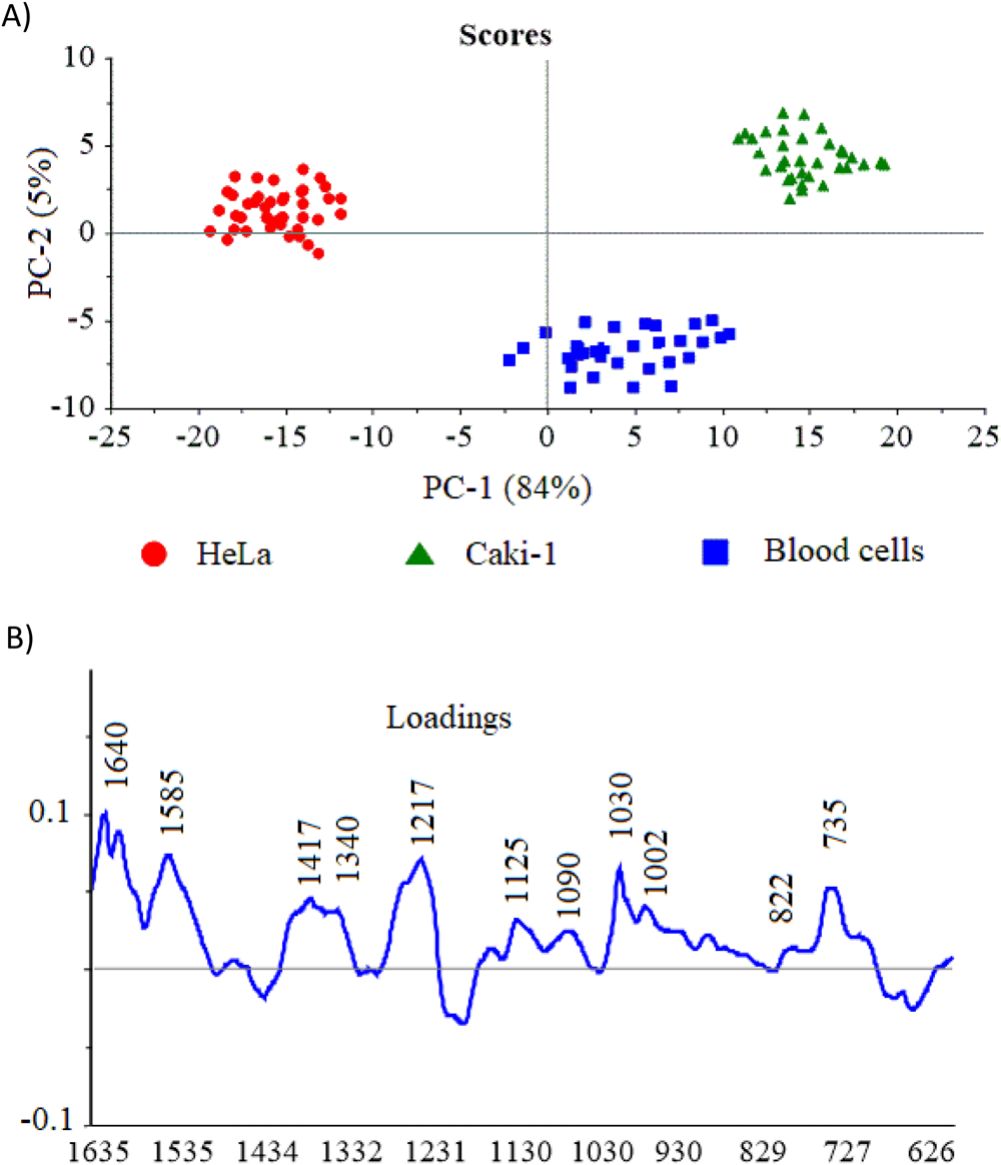
(**A**) The plots of PC-1 versus PC-2 scores component calculated for blood cells, HeLa and Caki-1 cells and corresponding PC-1 loading data (**B**).

PCA is wildly used multivariate technique, in which the large data set is defined into a new set of orthogonal variables represented by only a few factors called the principal component scores (PCs). In our studies the data sets including 120 spectra obtained for different cell types (HeLa, Caki-1, and blood cells captured after CTCs isolation) were analyzed by PCA. The independent-sample *t* test performed on all principal component scores shows that only two PCs (PC-1 and PC-2) are important (p < 0.005) for analyzed cells differentiation. The PC-1 and PC-2 were accounted in the wavenumber region 600–1660 cm^−1^, and explained the variance of 89%. In this way, these two PCs allow the effective differentiation between all studied cells (see Fig. 8, which illustrates the use of PC scores 1 and 2 for the classification of cells).

A score plot of PC-1 to PC-2 (Fig. 8A) shows that all SHINERS data could be divided into three groups (clusters) corresponding to blood cells (blue), renal cell carcinoma (Caki-1) (blue) and HeLa (red). The cluster of blood cells shows the relatively lower homogeneity in comparison to cancer cells, which is related with more complex structural changes of all expected cells in filtrated blood e.g. erythrocytes and lymphocytes. The spectral differences within the HeLa and Caki-1 clusters can be also observed and might be assignment to structural changes of biomolecules and down or up-regulation processes in cancer cells^67^.

It should be highlighted, that between samples fitting to one type of cells (the same group of classification) there are not diagnostically significant differences. The samples are well grouped together. However, the three different groups of cells (clusters) are spread in the large distances among them what additionally points out the excellent group differentiation.

The SHINERS spectra (Fig. 4) together with loading of PC-1 (Fig. 8B) give insight into the variables (wavenumber) that are the most significant for the group (cells) differentiations and provide the qualitative information about their molecular structure. As can be seen in Fig. 8B, the wavenumber of ca. 735 cm^−1^ (thiocyanate) 1030 cm^−1^ (phenylalanine and/or lipid), 1217 cm^−1^ (amide III), 1340 cm^−1^ (proteins, glutamic acid, serine, methionine, histidine), 1417 cm^−1^ (C = C stretching in quinoid ring), 1585 cm^−1^ (phenylalanine, tyrosine) and 1640 cm^−1^ (amide I) have the largest weights in the variations and point the most important differences between analysed cells. There are other wavenumbers at *ca*. 822, 1090, 1125 cm^−1^ which also have the significant contribution to PC-1 in the same direction as the most intensive loadings.

The appearance the band at 822, 1125, 1217 and 1417 cm^−1^ in cancer cells has been previously reported^68–71^.

As can be seen, many spectral bands calculated by PCA are consistent with SHINERS features presented in Fig. 6. This result illustrates that all studied cells are clearly separated into three clusters matching to the renal cell carcinoma (Caki-1), cervical carcinoma (HeLa) and blood cells, respectively.

Moreover, based onto the calculated the PCs, the R-square (R^2^) factor, which gives information about the sensitivity of used model has been assumed. The R^2^ factor was calculated for the optimal number of the principal components in analysed data sets and finally equals 0.998. Such excellent sensitivity demonstrates the great potential of SHINERS analysis combined with developed PCA method for label-free detection of tumor cells.

## Conclusions

In this work we present an efficient, non-invasive, and label-free method to isolate and structural and biochemical analysis of circulating tumor cells (CTs) in blood samples. The strength of reported SERS-based detection lies in merging:

(i) a SERS-active platform based on photovoltaic cell (Ag/PV) that offers high sensitivity, reproducibility and stability of recorded SERS signals,
(ii) an appropriately designed SHINs (shell-isolated nanoparticles) in the form of silica coated silver nanoparticles that additionally improved SERS sensitivity,
(iii) the constructed microfluidic device for high-throughput isolation of cancer cells from whole blood samples, and
(iv) the principal component analysis (PCA) applied to develop the diagnostic algorithms for improving the efficient screening, detection, and discrimination of a particular CTCs in complex biological samples.

The established statistical model achieved diagnostic accuracy up to 89% for differentiation of blood cells, renal cell carcinoma (Caki-1) and cervical carcinoma (HeLa) cells, based on SHINRS spectra of individual cells.

In future, the developed device for CTCs separation and analysis will be adopted to the investigation of other types of cancer biomarkers from clinical materials. Our approach with all its advantages has huge potential for developing the personalized cancer treatment.

### Ethical statements

All experiments were performed in compliance with the relevant laws and institutional guidelines. The protocol of study was approved by the Ethics and Bioethics Committee of Cardinal Stefan Wyszynski University in Warsaw. Informed consent was obtained from all patients.

## Supplementary information

Detection of circulating tumor cells in blood by shell-isolated nanoparticle – enhanced Raman spectroscopy (SHINERS) in microfluidic device
