# Supplementary material for: Detection of circulating tumor cells in blood by shell-isolated nanoparticle – enhanced Raman spectroscopy (SHINERS) in microfluidic device
Source: Sci Rep. 2019 Jun 25;9:9267. doi: 10.1038/s41598-019-45629-7 (PMC6592934; doi:10.1038/s41598-019-45629-7)
Supplement: Supplementary file 1 — Detection of circulating tumor cells in blood by shell-isolated nanoparticle – enhanced Raman spectroscopy (SHINERS) in microfluidic device [file 41598_2019_45629_MOESM1_ESM.docx]

**Supplementary Materials**

**Detection of circulating tumor cells in blood by shell-isolated nanoparticle – enhanced Raman spectroscopy (SHINERS) in microfluidic device**

K. Niciński^a^ , J. Krajczewski ^b^ A. Kudelski^b^, E. Witkowska^a^, J. Trzcińska-Danielewicz^c^, A. Girstun^c^, and A. Kamińska^a^*

^a^Institute of Physical Chemistry, Polish Academy of Sciences, Kasprzaka 44/52, 01-224 Warsaw, Poland

^b^Faculty of Chemistry, University of Warsaw, Pasteura 1, 02-093 Warsaw, Poland

^c^Department of Molecular Biology, Institute of Biochemistry, Faculty of Biology, University of Warsaw, 02-096 Warsaw, Poland

**1 Characterization PV surfaces**

| **Ion etched Si** | |
| --- | --- |
| **A)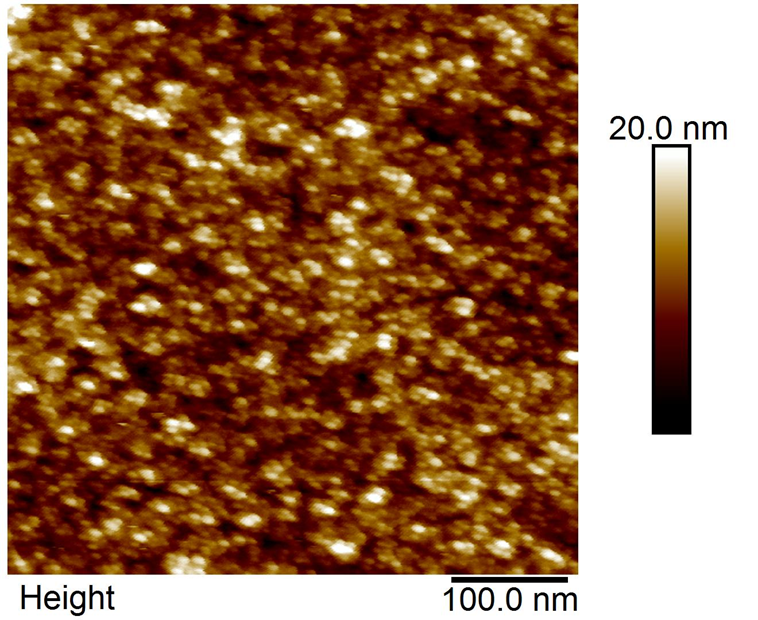**  **Roughness (*R*_a_): 6.6 ± 0.6 nm** | 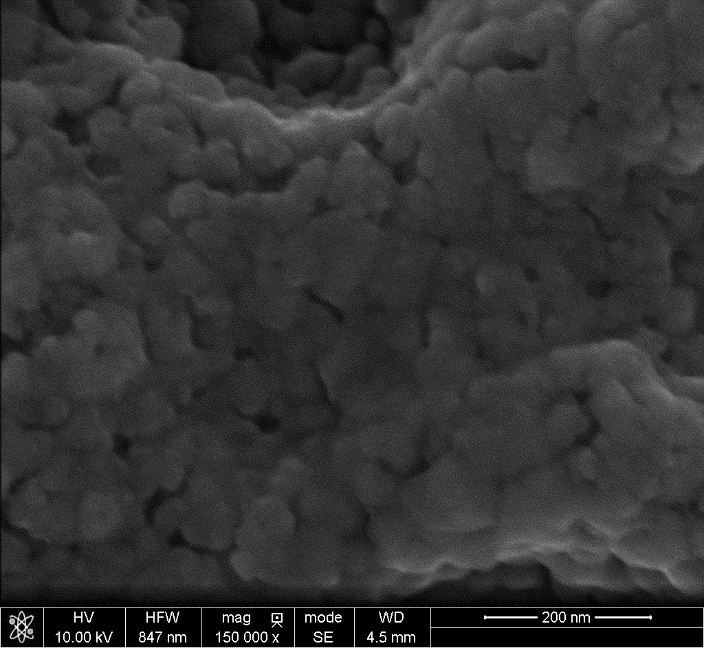**B)** |

**Fig. S1**. (A) AFM and (B) SEM images of ion etched silicon covered with silver layer[1].

The SERS spectral features of *p*-ATP adsorbed from 10^-6^ M ethanol solution onto four obtained Ag/PV surfaces were recorded and presented n Fig. S3. The two a_1_-typy bands at 1078 and 1593 cm^−1^ appearing also in the SERS spectra. The week band observed in normal Raman spectrum at 1172 cm^-1^ appears also in the SERS spectrum at 1180 cm^-1^. But, in contrast to normal Raman spectrum, the intensive b_2_-type bands (out of plane vibrational modes) at 1370, 1145, have been observed. The appearing of b_2_-type bands is related with contribution of CT mechanism (metal – molecule charge transfer process) to total SERS enhancement[2]. Moreover, the strong enhancement of these bands indicate the perpendicular orientation of the *p*-ATP molecules onto the Ag/PV surfaces[3].

To gain the further inside into the SERS performance the enhancement factor (EF) for Ag/PV SERS-active surface has been calculated using equation (1).

| EF= (I_SERS_/N_SERS_) / (I_NR_/N_NR_) | (1) |
| --- | --- |

where *N*_SERS_ and *N*_NR_ refer to the number of molecules adsorbed on the SERS probe within the laser spot area and the number of molecules probed by regular Raman spectroscopy, respectively. *I*_SERS_ and *I*_NR_ correspond to the SERS intensity of *p*-ATP on the modified surface and to the normal Raman scattering intensity of *p*-ATP in the bulk. *I*_NR_ and *I*_SERS_ were measured at 1078 cm^-1^. From these data of the relative intensity and the number of molecules sampled from the regular Raman and SERS measurements, the enhancement factors have been calculated at the 10^6^ level.


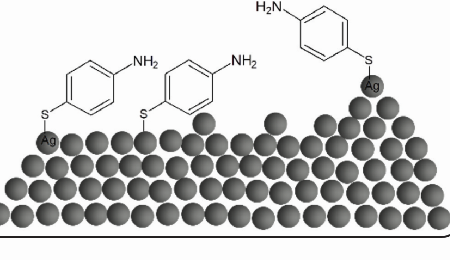

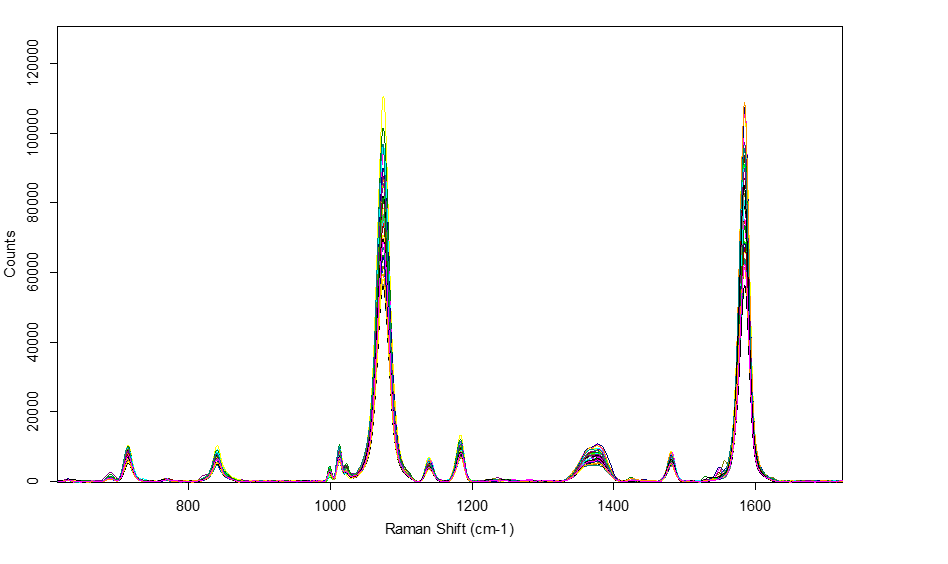


**Fig. S2.** The representative SERS spectra *p*-ATP of concentration 10^-6^ M in ethanol recorded from 40 different spots on the Ag/PV SERS-active surface using mapping mode. The spectra were collected over an area 10 x 20 µm with 1.6 steps µm (40 spectra are shown). Each point in the map was recorded using 1.5 mW of 785 nm excitation with 18 seconds integration times.

**2. SERS results**

|  |
| --- |
| (b)   |

**Fig. S3.** Reference SERS spectra of (a) HeLa and (b) Caki-1cells placed directly from pre-cultures onto SERS substrate.

**Table S1.** Observed vibrational bands of whole human blood (liquid droplet) at 785 nm and their assignments[4-7]. Vibrations: n – valence, d – deformation, g – deformation (out of plane).

| **Observed SERS band** | **Vibrational mode** | **Assignment** |
| --- | --- | --- |
| 638 | C-S stretch | globin and cellular components (cysteine) |
| 716 | d(COO^-^) | globin and cellular components (amino-acids) |
| 796 | n(pyr breathe), n_6_ | porphyrin |
| 863 | g(C_m_H) | porphyrin |
| 904 | C-C stretch | globin and cellular components (glutamic acid, isoleucine, threonine, lysine) |
| 956 | C-C stretch | globin and cellular components (proteins) |
| 1002 | indole assymetric ring breathe | globin and cellular components (phenylalanine) |
| 1027 | in plane ring CH deform | globin and globin and cellular components (phenylalanine) |
| 1104 | C-N, C-C stretch | globin and cellular components (proteins, lipids) |
| 1127 | C-N, C-C stretch | globin and cellular components (proteins) |
| 1215 | d(C_m_H), n_13_^,^or n_42_ | porphyrin |
| 1257 | CH_2_ wagging  d(CH_2_/CH_3_)_6_ | globin (glutamic acid) and cellular components (proteins, lipids: amide III) |
| 1286 | g(C_m_H), n_21_ | porphyrin |
| 1326 | CH_2_ wagging | globin and cellular components (phenylalanine, glutamic acid, serine, methionine, histidine) |
| 1342 | CH2 scissoring  CH3 deformation | globin and cellular components -glutamic acid, aspartic acid, asparagine, glutamine;  -alanine, leucine, valine, isoleucine |
| 1446 | d(CH_2_/CH_3_) | globin and porphyrin |
| 1488 | CH and ring | tryptophan |
| 1562 |  | porphyrin (skeletal mode) and cellular components |
| 1609 |  | porphyrin |

**3. Reproducibility of the SERS – based method of CTC detection.**

The reproducibility of the recorded SERS signals plays a crucial role in the analytical and biomedical applications of SERS technique. The relative standard deviation (RSD) of the SHINERS signals of HeLa and Caki-1 cells were calculated and presented in the Table S2.

**Table S2.** The RSD of the selected intensities of SHINERS signals of HeLa and Caki-1 cells recorded from 100 different spots within the same sample.

| **Cell type** | **Selected bands [cm^-1^]** | **RSD (%)** |
| --- | --- | --- |
|  |  |  |
| HeLa | 1613 | 8.5 |
| Caki-1 | 658 | 7.2 |

References:

[1] K. Niciński, E. Witkowska, D. Korsak, K. Noworyta, J. Trzcińska-Danielewicz, A. Girstun, A. Kamińska, Photovoltaic cells as a highly efficient system for biomedical and electrochemical surface-enhanced Raman spectroscopy analysis, RSC Advances, 9 (2019) 576-591.

[2] X. Hu, T. Wang, L. Wang, S. Dong, Surface-Enhanced Raman Scattering of 4-Aminothiophenol Self-Assembled Monolayers in Sandwich Structure with Nanoparticle Shape Dependence:  Off-Surface Plasmon Resonance Condition, The Journal of Physical Chemistry C, 111 (2007) 6962-6969.

[3] M. Moskovits, J.S. Suh, Surface selection rules for surface-enhanced Raman spectroscopy: calculations and application to the surface-enhanced Raman spectrum of phthalazine on silver, The Journal of Physical Chemistry, 88 (1984) 5526-5530.

[4] S. Stewart, P.M. Fredericks, Surface-enhanced Raman spectroscopy of amino acids adsorbed on an electrochemically prepared silver surface, Spectrochimica Acta Part A: Molecular and Biomolecular Spectroscopy, 55 (1999) 1641-1660.

[5] N.A. Brazhe, S. Abdali, A.R. Brazhe, O.G. Luneva, N.Y. Bryzgalova, E.Y. Parshina, O.V. Sosnovtseva, G.V. Maksimov, New insight into erythrocyte through in vivo surface-enhanced Raman spectroscopy, Biophys J, 97 (2009) 3206-3214.

[6] J.L. Lippert, L.E. Gorczyca, G. Meiklejohn, A laser Raman spectroscopic investigation of phospholipid and protein configurations in hemoglobin-free erythrocyte ghosts, Biochim Biophys Acta, 382 (1975) 51-57.

[7] D.F. Wallach, S.P. Verma, Raman and resonance-Raman scattering by erythrocyte ghosts, Biochim Biophys Acta, 382 (1975) 542-551.
